# Literary Note

**Published:** 1904-09

**Authors:** 


### LITERARY NOTE.

THE *Medical Book News*, published by P. Blakiston's Son & Company, Philadelphia, with the issue for July, 1904, makes the change from a bi-monthly to a monthly periodical. New departments have been added, illustrations introduced and other improvements made that will tend to increase the general worth and utility of this magazine. The subscription price has been raised from 25 cents to 50 cents a year.

